# Fully-automated multi-objective optimization for fitting a neuronal model with real morphology

**DOI:** 10.1186/1471-2202-16-S1-P117

**Published:** 2015-12-18

**Authors:** Aushra Abouzeid, Nelson Spruston, William Kath

**Affiliations:** 1 Engineering Sciences and Applied Mathematics, Northwestern University, Evanston, IL 60208, USA; 2 Howard Hughes Medical Institute, Janelia Research Campus, Ashburn, VA 20147, USA

## 

Morphologically realistic models have successfully been used to elucidate many complex mechanisms in neuronal dendrites. However, the tuning of such models to match experimental data remains challenging. Here we introduce a fully automated parameter optimization methodology that uses the Python programming language to control the NEURON simulator in parallel on a high performance computing cluster.

Using targeted experimental protocols, including sub- and supra-threshold somatic as well as dendritic voltage recordings, we constrain a model hippocampal CA1 pyramidal cell built with a complete reconstructed morphology. The optimization is performed using the non-dominated sorting genetic algorithm (NSGA-II), and model fitness is evaluated by directly comparing the simulated and recorded voltage traces. In order to impose minimal a priori assumptions, we use a multi-objective framework, which tunes all of the free parameters with respect to all of the experimental objectives simultaneously. Furthermore, the multi-objective approach avoids the pitfalls of overfitting, because the algorithm produces a diverse family of solutions on the so-called Pareto-optimal front. To facilitate model selection, we have developed a clickable interface for visually browsing the set of optimal solutions, which permits the explicit and rapid identification of trade-offs among the fitting objectives and the biophysical parameters that govern variability in the solution set.

**Figure 1:**
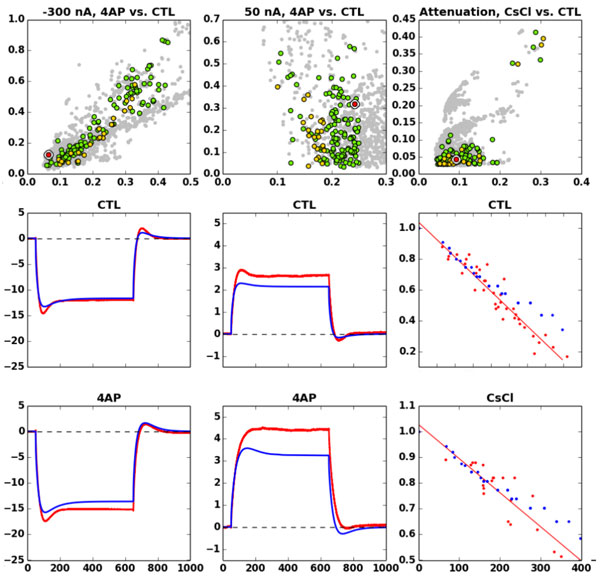
**Clickable interface for browsing the Pareto front of solutions produced by our multiobjective optimization platform**. Top row: Scatterplot of error scores across a 6-dimensional error space. The abscissa and ordinal represent errors in the objectives shown below in the same column. A highlighted point appears in each scatterplot (white circle with red center) to indicate errors produced by a single model instance across each of the six objectives. Bottom two rows: Experimental traces (red) and model output (blue). The right-most column depicts dendritic voltage attenuation as a function of distance from soma as measured in dual patch-clamp recordings along the main apical dendrite.

